# Phenomenology of psychiatric stigma: A factor of patients’ motivation to treatment

**DOI:** 10.1192/j.eurpsy.2021.1344

**Published:** 2021-08-13

**Authors:** M. Sorokin, N. Lutova, V. Wied

**Affiliations:** The Integrative Pharmaco-psychotherapy Of Mental Disorders, V.M.Bekhterev National medical research center for psychiatry and neurology, Saint-Petersburg, Russian Federation

**Keywords:** Stigma, motivation, adherence

## Abstract

**Introduction:**

Psychiatric patients often are self-stigmatized and hardly involve in the treatment.

**Objectives:**

Associations of self-stigmatizing beliefs in psychiatric inpatients and their treatment motivation.

**Methods:**

63 inpatients; ICD-10: F2–65%, F3–13%, F4+F6–14%, F06–8%; mean age 34±13, illness duration 12±11 years. Treatment Motivation Assessment Questionnaire (TMAQ), Internalized Stigma of Mental Illness scale (ISMI); K-mean cluster analysis; dispersion analyses; p≤0.05.

**Results:**

18 patients of cluster 1 (C1) demonstrated explicit self-stigmatization. In comparison with 25 subjects from cluster 3 (C3) stigmatized patients (C1) had higher levels of overall ISMI scores (2.9±0.3) caused by alienation (3.1±0.5), stereotype endorsement (2.5±0.5), social withdrawal (2.7±0.4), and discrimination experience (2.7±0.4). 20 patients of cluster 2 (C2) had an implicit stigma. They were more self-stigmatized (ISMI score 2.7±0.3) in contrast with subjects from cluster 3 (1.9±0.2) due to a lower level of stigma resistance (C2: 3.8±0.5 and C3 3.1±0.6 – reverse scores). Patients with implicit self-stigma (C2) had the lowest intensity of treatment motivation (Z-scores -1.2±0.6) compering with others (C1 and C3) due to the lowest TMAQ factor 1 (reliance on own knowledge and skills to cope with the disorder: -1.0±0.6) and factor 4 (willingness to cooperate with doctor: -0.9±1.0). Differences between explicitly and implicitly stigmatized patients manifested also in lower TMAQ factor 3 for the second group (awareness of the psychological mechanism of maladaptation: -0.5±0.9).

**Conclusions:**

Despite alienation, stereotype endorsement, social withdrawal, discrimination experience some patients could sustain stigma due to cooperation with doctors and reliance on their own knowledge and skills to cope with illness.

